# Research on Intelligent Wood Species Identification Method Based on Multimodal Texture-Dominated Features and Deep Learning Fusion

**DOI:** 10.3390/plants15010108

**Published:** 2025-12-30

**Authors:** Yuxiang Huang, Tianqi Zhu, Zhihong Liang, Hongxu Li, Mingming Qin, Ruicheng Niu, Yuanyuan Ma, Qi Feng, Mingbo Chen

**Affiliations:** 1 College of Materials and Chemical Engineering, Southwest Forestry University, Kunming 650224, China; yxhuang@swfu.edu.cn (Y.H.); 13085315910@163.com (H.L.); swfuqmm@swfu.edu.cn (M.Q.); 2 College of Big Data and Intelligent Engineering, Southwest Forestry University, Kunming 650224, China; tiks39862@gmail.com (T.Z.); nrc@swfu.edu.cn (R.N.); mayy@swfu.edu.cn (Y.M.); fengqi@swfu.edu.cn (Q.F.); chen2239147428@gmail.com (M.C.)

**Keywords:** wood cross-section, hyperspectral image, ST-former, multimodal fusion, complementary collaborative learning

## Abstract

Aimed at the problems of traditional wood species identification relying on manual experience, slow identification speed, and insufficient robustness, this study takes hyperspectral images of cross-sections of 10 typical wood species commonly found in Puer, Yunnan, China, as the research object. It comprehensively applies various spectral and texture feature extraction technologies and proposes an intelligent wood species identification method based on the fusion of multimodal texture-dominated features and deep learning. Firstly, an SOC710-VP hyperspectral imager is used to collect hyperspectral data under standard laboratory lighting conditions, and a hyperspectral database of wood cross-sections is constructed through reflectance calibration. Secondly, in the spectral space construction stage, a comprehensive similarity matrix is built based on four types of spectral similarity indicators. Representative bands are selected using two Max–Min strategies: partitioned quota and coverage awareness. Multi-scale wavelet fusion is performed to generate high-resolution fused images and extract interest point features. Thirdly, in the texture space construction stage, three types of texture feature matrices are generated based on the PCA first principal component map, and interest point features are extracted. Fourthly, in the complementary collaborative learning stage, the ST-former model is constructed. The weights of the trained SpectralFormer++ and TextureFormer are imported, and only the fusion weights are optimized and learned to realize category-adaptive spectral–texture feature fusion. Experimental results show that the overall classification accuracy of the proposed joint model reaches 90.27%, which is about 8% higher than that of single-modal models on average.

## 1. Introduction

Wood has always been one of the essential renewable materials in people’s lives [1]. Due to its natural characteristics such as beautiful texture and color, good elasticity, light weight, and easy processing, wood plays a pivotal role in fields such as structural materials, decoration, and energy fuels and is one of the main substrates in people’s production and life [2,3,4]. However, driven by interests, counterfeiting and shoddy phenomena often occur in the wood trading process. This behavior of passing off inferior products as high-quality ones and passing off fake products as genuine ones has brought very serious negative impacts on the wood trading market [5,6,7]. At the same time, wood illegally logged accounts for as high as 10% of the total wood cut worldwide, causing great damage to the environment [8]. To protect the legitimate rights and interests of consumers, the use of convenient and accurate intelligent methods for wood species identification has become one of the research directions of many scholars [9].

Wood species identification methods are mainly divided into macro and micro categories. Macro identification judges the species through the color, texture, and smell of wood. Although this method is simple, it is prone to misjudgment because the features may not be significant or may be easy to counterfeit. Micro identification identifies tree species by means of the microscopic structure of wood (such as pore characteristics, wood ray characteristics, etc.). According to the different magnification, it can be further divided into high-magnification identification and low-magnification identification [10,11,12,13]. In recent years, innovative technologies such as near-infrared spectroscopy (NIRS), isotope and mineral element methods, and DNA molecular markers have provided important approaches for wood species identification [14].

As an analytical technology based on molecular vibration and rotational energy level transitions, NIRS achieves qualitative and quantitative analysis of substance components by detecting the absorption spectrum of molecules in the near-infrared region [15,16]. Richardson et al. [17] collected near-infrared spectral data of wood powder from different regions and converted the data with a mathematical relationship calibration model, which realized the spectral conversion between intact wood and wood powder. Zhuang et al. [18] conducted a feasibility study on the origin traceability of *Pterocarpus santalinus* based on peak and valley feature extraction. The main component content of the same wood from different origins is different. The vibrational absorption of hydrogen-containing groups in the near-infrared spectrum can reflect this difference information. The experimental results show the high sensitivity and accuracy of this method. Raobelina et al. [19] extracted key spectral feature variables of wood samples and used principal component analysis to reduce the dimensionality of the original high-dimensional wavenumber variables, which significantly reduced the number of wavenumber variables involved in modeling. Experimental results show that compared with traditional discriminant models, this method greatly reduces the model complexity while maintaining high classification accuracy, providing a reliable analytical method for wood species identification. Peng et al. [20] used three methods to preprocess the spectrum to eliminate interference information and then used a variety of machine learning algorithms to achieve good stability of the neural network model and high accuracy of wood species identification.

As a natural organic material, wood experiences isotope fractionation during its growth process under the combined effects of climatic conditions, ecological environment, and metabolic activities in organisms [21]. This fractionation phenomenon is manifested in significant differences in the abundance of isotopes from different pathways in wood [22]. Chen et al. [23] proposed that C3 and C4 plants have different fractionations when fixing CO_2_ through photosynthesis and are affected by environmental factors (such as drought and light intensity), thereby distinguishing wood from arid and humid areas (stomatal closure of plants under drought conditions leads to enrichment). Landry et al. [24] showed that oxygen isotopes (δ^18^O) reflect the isotopic composition of water sources (precipitation, groundwater), which is affected by temperature, altitude, and latitude, distinguishing wood from different river basins or altitudes. He et al. [25] revealed that plant water comes from precipitation, which is related to latitude and the continental effect. Tonouewaj et al. [26] confirmed, through the study of nearly 1000 wood samples collected from different regions such as Cameroon, Congo, Gabon, and Indonesia, that the elemental fingerprint method is highly feasible and accurate in distinguishing the origin of wood at national and even more refined regional scales. Kanbayashi et al. [27] adopted inductively coupled plasma mass spectrometry (ICP-MS) technology to carry out high-sensitivity detection and analysis of trace elements. Stukonyte et al. [28] proposed a rapid non-destructive testing method for wood based on X-ray fluorescence spectroscopy (XRF). Hao et al. [29] proposed an analytical method based on laser ablation inductively coupled plasma mass spectrometry (LA-ICP-MS), which can realize high-precision and high-resolution detection of the distribution characteristics of mineral elements in tree growth rings.

The DNA of different wood species shows significant similarity on the whole. It is these seemingly small differences that provide an effective way to distinguish woods that are almost indistinguishable in structure and appearance. Yan et al. [30] proposed a more refined wood classification method, aiming to break through the limitation of the traditional classification system that only stays at the “genus” level. Tran et al. [31] focused on selecting three DNA barcode sequences with important identification value, namely, rpoC1, trnH-psbA, and ITS, as molecular markers, established a complete molecular biological detection system, and conducted scientific and accurate traceability identification of the origin information of wood samples. Mo et al. [32] designed and constructed a wood species traceability system based on high-resolution barcode technology, highlighting the application value of DNA barcode technology in the field of wood identification. Ajdary et al. [33] constructed a wood DNA fingerprint database by analyzing the unique DNA sequence polymorphism, single nucleotide polymorphisms (SNPs), and microsatellite markers of wood samples, combined with bioinformatics analysis and machine learning algorithms, so as to realize the accurate identification of different woods.

The advantages and disadvantages of the commonly used methods for identifying wood species are shown in Table 1.

**Figure 1:**
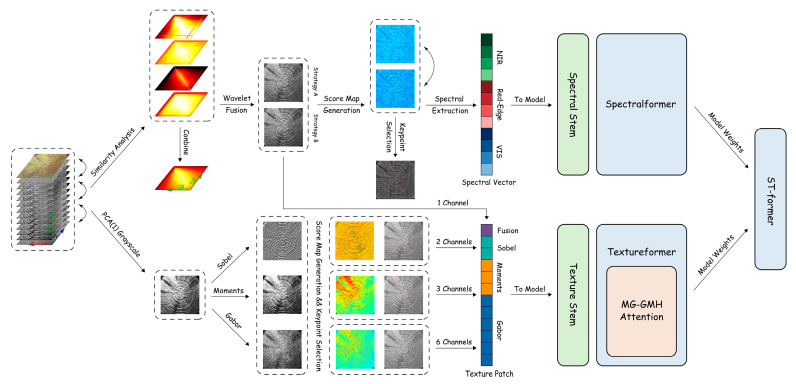
Overall flowchart of the research methodology.

In view of the above problems, this study proposes an intelligent wood species identification method based on the fusion of multimodal texture-dominated features and deep learning. The overall flow of the research method in this paper is shown in Figure 1. Firstly, the SOC710-VP hyperspectral imaging instrument was used to collect hyperspectral data under standard laboratory lighting conditions, and a wood cross-sectional hyperspectral database was constructed through reflectance calibration. In the spectral space construction stage, two Max–Min strategies are used to screen representative bands, and multi-scale wavelet fusion is performed to generate high-resolution fused images. In the texture space construction stage, three types of texture feature matrices are generated based on the PCA first principal component map. In the single model training phase, spectral branch SpectralFormer++and texture branch TextureFormer are constructed, and a multi-mode interest point guidance mechanism is introduced to realize fine modeling and feature complementation in texture domain. Finally, in the complementary collaborative learning stage, the ST former model is constructed, and the trained SpectralFormer++and TextureFormer weights are imported. Only the fusion weights are optimized to achieve category-adaptive spectral–texture feature fusion.

## 2. Data Collection and Preprocessing

### 2.1. Wood Samples

This study selects hyperspectral images of cross-sections of 10 different tree species widely distributed in Puer, Yunnan, China, as the research objects. These samples are collected from different tree bodies to ensure the diversity and representativeness of the research data.

### 2.2. Collection Method

**Figure 2:**
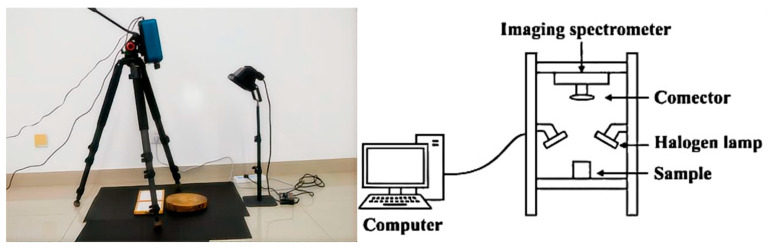
Instrument placement diagram.

The specific steps are as follows. First, a circular saw is used to cut along the cross-section from the breast height of the tree (about 1.3 m high) to form a cylindrical wood sample with a thickness of about 30 cm. Subsequently, an SOC710-VP hyperspectral imager and a microscope are used to collect hyperspectral images of the selected samples, and the magnification of the microscope is set to 40–50×. Figure 2 shows the completed image collection environment.

### 2.3. Dataset Preprocessing

**Figure 3:**
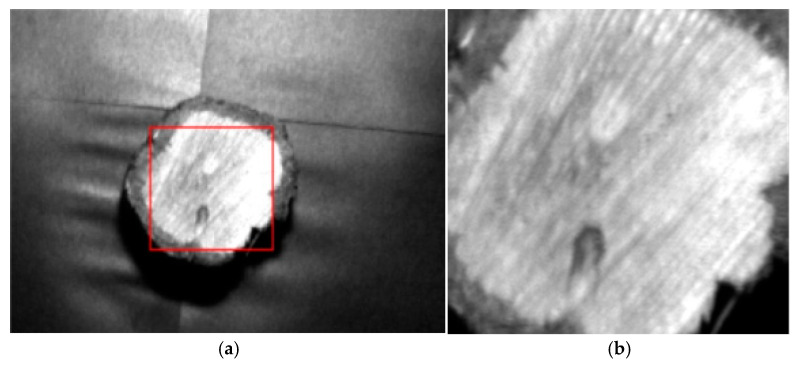
Comparison between the original image and the cropped image: (**a**) original image (band 42); (**b**) image after cropping (band 42).

**Figure 4:**
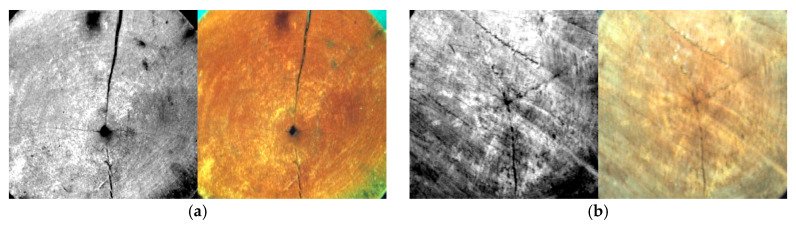

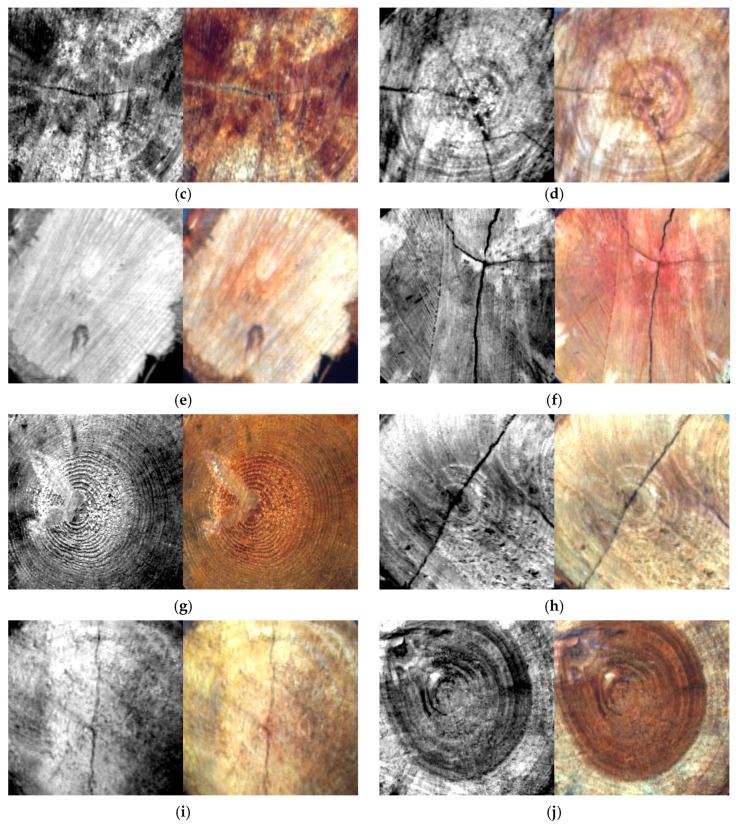
Hyperspectral and corresponding RGB images of 10 tree species samples: (**a**) *Ailanthus altissima* (Mill.) Swingle; (**b**) *Betula alnoides* Buch.-Ham. ex D. Don; (**c**) *Eucalyptus rudis* Endl; (**d**) *Senna siamea* (Lam.) H. S. Irwin & Barneby; (**e**) *Quercus aliena* Blume; (**f**) *Phoebe rufescens* H. W. Li; (**g**) *Pinus kesiya var. langbianensis* (A. chev.) Gaussen; (**h**) *Betula platyphylla* Sukaczev; (**i**) *Fraxinus malacophylla* Hemsl; (**j**) *Cinnamomum parthenoxylon* (Jack) Meisn.

Since the samples have circular cross-sections, the acquired images display rectangular frames, and the four-corner areas usually do not contain effective wood cross-sectional texture information, which is likely to introduce background noise. To ensure the reliability and practicality of the experimental data, ENVI Classic 5.6 is used to crop the collected wood cross-section images to the inscribed rectangular region of the wood cross-section. Figure 3 shows a comparison between the original image (42 bands) and the cropped image (42 bands). Figure 4 presents the collected hyperspectral images of the 10 wood species samples and their corresponding RGB images.

## 3. Methods and Analysis

In digital image processing, the structure of image data exhibits a significant hierarchical organization depending on the image type [34]. During hyperspectral image processing, each pixel at a given spatial location contains complete spectral information, manifested as a spectral reflectance curve composed of L discrete sampling points. This curve accurately characterizes the spectral properties of the spatial location across different wavelengths [35,36,37].

### 3.1. Multimodal Texture-Dominated Spectral Space Construction

#### 3.1.1. A Hyperspectral Band Selection Method Based on Four Types of Indicators

Before processing hyperspectral image data, dimensionality reduction is usually required. Currently, dimensionality reduction methods for hyperspectral images are mainly divided into two categories: feature extraction and feature selection. In terms of feature extraction, the main algorithms include principal component analysis (PCA), Fisher Linear Discriminant Analysis (FLD), and Minimum Noise Fraction (MNF) [38,39,40].

To comprehensively consider the image features of wood hyperspectral images, this study proposes a hyperspectral band selection method based on four categories of indicators. Compared with traditional methods, this method shows significant advantages in subsequent feature extraction and classification. To further investigate the characteristics of the 3028 cropped images, this study conducted a band similarity analysis on a hyperspectral cube consisting of 128 bands for each sample. Four analytical techniques were employed in the analysis, namely, Fast Frequency Domain Differential Mapping (FDDM), Difference Hash Similarity (DHashM), Mutual Information (MIM), and Structural Similarity Index (SSIM). The integrated use of these techniques aims to provide a detailed description and evaluation of the differences between bands from multiple dimensions, ensuring the accuracy and completeness of the analysis results.

To address the inconsistency in value scales among different indicators, the similarity results of each indicator are first normalized, and a comprehensive similarity matrix $S_{ij}^{comb}$ is then constructed through weighted fusion to characterize the overall similarity between any two spectral bands.

After obtaining the comprehensive similarity matrix $S_{ij}^{comb}$, a multi-strategy band selection mechanism is introduced. This mechanism includes two forms: Strategy A (regional quota + Max–Min) and Strategy B (coverage reward Max–Min), to ensure that the selected bands cover the visible, red, and near-infrared regions, while achieving a balance between information redundancy and diversity.

(1) Strategy A: Partition quota and maximum–min distance method (Max–Min)

First, based on the wavelength range calibrated by the spectrometer (372.53–1038.57 nm), the entire spectrum is divided into three segments: the visible light region (VIS), the red light region (RED), and the near-infrared region (NIR). Then, based on the number of bands and their distribution characteristics in each segment, a quota ratio of 3:3:4 is set, and within each segment, several bands are selected using the maximum–min distance method (Max–Min).

Let the comprehensive difference matrix $\Delta_{ij}=1-S_{ij}^{comb}$, and the selected band set be Ω. Then, in each iteration, the band that satisfies the condition of having the largest minimum distance is selected:

(1) $$b^{*}=arg\;\underset{b\notin \Omega }{max}\;\underset{s\in \Omega }{min}\;\Delta_{b,s}$$

This continues until the set number of bands k is reached. This strategy effectively avoids redundancy caused by excessively dense bands and ensures the representativeness of each spectral segment.

(2) Strategy B: Coverage-aware Max–Min method

To further improve the coverage of the selected bands across the three major spectral ranges, a coverage reward factor $\lambda_{cov}$ is introduced, and a scoring function is defined:

(2) $$score(b)={min}_{s\in \Omega }\Delta_{b,s}\times \left\{\begin{array}{ll} 1+\lambda_{cov}, & if\;r(b)\notin C,\\ 1, & if\;r(b)\in C. \end{array}\right.$$

where r(b) denotes the spectral region (VIS, RED, or NIR) to which band b belongs, and C denotes the set of spectral regions that have already been covered by the selected band set.

#### 3.1.2. Band Fusion Method Based on Wavelet Transform

After obtaining the most representative set of bands from the spectral data of each sample, this study designs a multi-band fusion algorithm based on multi-scale wavelet transform to further integrate the complementary information contained in different bands, performing joint spatial and frequency domain optimization of the spectral information.

Let the hyperspectral cube obtained after band filtering be $I_{band}\in R^{B\times H\times W}$, where B represents the number of bands. Based on the band index sets obtained from strategies A and B, the corresponding band images $\{I_{band,b}{\}}_{b=1}^{N_{band}}$ are extracted, where $N_{band}=10$. This study uses the Daubechies-4 wavelet basis function and sets the decomposition level L=2 to ensure a balance between resolution and stability.

#### 3.1.3. Spectral Feature Extraction

After successfully acquiring wavelet fusion images, this study employs an interest point detection algorithm based on frequency domain demeshing suppression and multi-feature fusion scoring to extract the most representative texture regions from wood cross-sectional images.

(1) Frequency domain notch degrid

Because periodic interference fringes often appear during the imaging process of experimental equipment, this paper first performs notch filtering on the input fused image $I_{fuse}(x,y)$ in the frequency domain.

The image is Fourier transformed into F(u,v), the grid peak center $\left(u_{i},v_{i}\right)$ is automatically estimated on the amplitude spectrum, and a Gaussian notch mask is constructed:

(3) $$M(u,v)=\prod_{i}\;\left[1-\alpha_{n}exp\left(-\frac{\left(u-u_{i}{)}^{2}+(v-v_{i}{)}^{2}\right.}{2\sigma_{n}^{2}}\right)\right]$$

where $\alpha_{n}$ is the suppression intensity (0.85) and $\sigma_{n}$ is the notch radius (3.5). After mask multiplication and inverse transformation, a smooth image $I_{dn}(x,y)$ is obtained, which effectively eliminates regular noise.

(2) Local enhancement and denoising

In the spatial domain, nonlocal means denoising (NL-Means, intensity 4.0) is employed, followed by joint enhancement using CLAHE (contrast-limited adaptive histogram equalization) and an anti-sharpening mask.

CLAHE adaptively enhances the brightness distribution using an 8×8 local mesh, with a clip limit of 2.0 to prevent excessive noise amplification. Subsequently, an anti-sharpening mask (radius 1.2, enhancement 1.3) and $\gamma_{h}$ correction $\left(\gamma_{h}=0.95\right)$ are applied to improve local texture contrast. For samples with uneven illumination, a homomorphic filtering mode can be selected, decomposing the logarithmic domain into low-frequency illuminance and high-frequency reflectance components and amplifying the reflectance term to correct brightness.

(3) Multi-feature scoring map construction

To comprehensively measure the texture saliency of local regions, a weighted scoring map $S_{kpt}(x,y)$ is defined:

(4) $$S_{kpt}=0.25N\left(Harris\right)+0.15N\left(Entropy\right)+0.15N\left(Sobel\right)+0.15N\left(Laplace\right)+0.15N\left(DoG\right)\;+0.15N\left(SNR\right)$$

where N(⋅) represents the linear normalization operation.

(4) Point of interest extraction

After selecting a multi-feature scoring map $S_{kpt}(x,y)$, a candidate point set is generated using Harris corner detection combined with local peak search. Let there be a relative threshold t=0.02 and a minimum spacing d=6. While ensuring uniform spatial distribution, the K=100 highest-scoring points are selected as the final interest points, sorted from highest to lowest significance value based on the scoring map.

### 3.2. Multimodal Texture-Dominated Texture Space Construction

To fully explore the differential information of wood cross-sectional texture structure, this paper establishes a two-dimensional feature space dominated by multimodal texture based on hyperspectral data. First, using the first principal component of the original hyperspectral image as the grayscale base image, three types of complementary texture features are extracted from it: Sobel edge features, second-order geometric moment features, and Gabor energy features.

(1) Principal component grayscale background image generation

The first principal component was extracted from the original hyperspectral cube $C\in R^{H\times W\times B}(B=128)$ using principal component analysis (PCA):

(5) $$I_{pca}={PCA}_{1}(C)$$

PCA employs a randomized singular value decomposition solver, retaining only the principal variance directions to obtain a standardized grayscale image $I_{pca}\in [0,1]$, which serves as the underlying image for texture feature extraction.

(2) Sobel edge features

The gradients are calculated in the horizontal and vertical directions using the Sobel operator:

(6) $$G_{x}=\frac{\partial I_{pca}}{\partial x},{\;G}_{y}=\frac{\partial I_{pca}}{\partial y}$$

After normalizing the gradient magnitudes in both directions, the gradients are stacked to form a two-channel edge feature map:

(7) $$F_{sobel}=[N(G_{x}),N(G_{y})]\in R^{H\times W\times 2}$$

(3) Second-order geometric moment characteristics

To characterize the local structural distribution of wood grain, a two-dimensional weight kernel of window size w=7 is constructed, and the second-order geometric moments are calculated:

(8) $$M_{20}=I_{pca}\times k_{20},\;{\;M}_{02}=I_{pca}\times k_{02},{\;M}_{11}=I_{pca}\times k_{11}$$

where

(9) $$k_{20}=x^{2},{\;k}_{02}=y^{2},{\;k}_{11}=xy$$

After convolution, the matrices are normalized and stacked to form a three-channel moment feature map:

(10) $$F_{moments}=[N(M_{20}),N(M_{02}),N(M_{11})]$$

This mode can reflect the anisotropy and geometric distribution of texture.

(4) Gabor energy characteristics

To characterize the texture frequency characteristics at different directions and scales, a Gabor filter $\theta \in \{0^{\circ },30^{\circ },60^{\circ },90^{\circ },120^{\circ },150^{\circ }\}$ with six directions is constructed, and its kernel function is defined as follows:

(11) $$g\left(x,y;\theta \right)=exp\left[-\frac{x^{\prime 2}+{\gamma_{g}}^{2}y^{\prime 2}}{2{\sigma_{g}}^{2}}\right]cos\left(2\pi \frac{x^{\prime }}{\lambda }\right)$$

where

$$x^{\prime }=xcos\;\theta +ysin\;\theta ,\lambda =8,\sigma_{g}=0.56\lambda ,\gamma_{g}=0.5$$

The filtered energy for each direction is normalized and stacked to form a six-channel feature map:

(12) $$F_{gabor}\in R^{H\times W\times 6}$$

This feature can capture directional, periodic textures such as wood rings and rays.

(5) Intramodal Interest Point Filtering and Saving

Calculate the comprehensive score matrix on each modal feature map (gradient magnitude for Sobel modes, L2 norm for moment modes, and maximum energy response for Gabor modes) and select the top K=100 salient points from each matrix.

### 3.3. Single Model Training

#### 3.3.1. Spectralformer++

**Figure 5:**
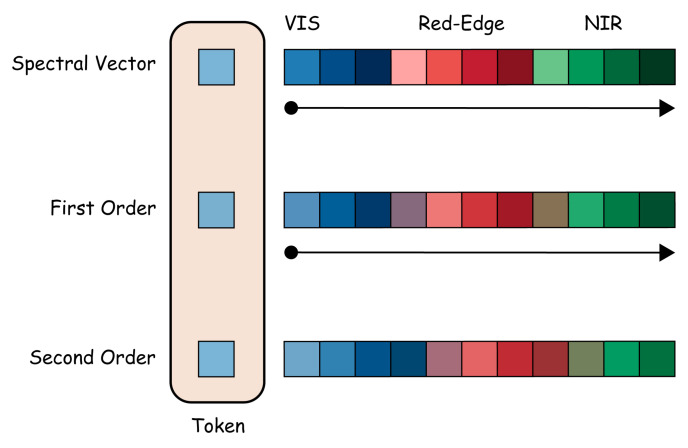
Spectral derivative prior modeling diagram.

To further improve the physical interpretability and classification robustness of wood spectral feature modeling, this paper proposes SpectralFormer++ based on the SpectralFormer model [41]. This model optimizes the input modeling and feature normalization based on the characteristics of spectral data while keeping the Transformer backbone unchanged, thereby significantly improving the model performance and stability. As shown in Figure 5, the spectral derivative prior is extended through the first-order and second-order difference spectral input channels, enabling the model to perceive changes in spectral morphology at the input stage, thus enhancing its ability to represent fluctuations in curvature, peak shape, and slope.

To further standardize the numerical scale of input features and stabilize the training process, SpectralFormer++ introduces a lightweight linear embedding layer on top of the differential multi-channel input, uniformly projecting the three-channel input into a 64-dimensional embedding space:

(13) $$E=LN(X^{sp}W+b),W\in R^{3\times d},d=64$$

Layer normalization is applied at the output to eliminate amplitude deviations between different bands. This structure enables the model to express the dynamic features of the spectrum at the input stage, simultaneously sensing the slope changes at the edges of the absorption band and the curvature features of the peak region, thereby more accurately distinguishing the differences in spectral response of different wood components.

#### 3.3.2. TextureFormer

To further enhance the spatial representation and structural robustness of wood cross-sectional texture features, this paper proposes the TextureFormer model based on the hierarchical shift window architecture of the Swin Transformer [42,43,44]. This model optimizes input modeling, front-end fusion, and attention guidance while maintaining the core hierarchical structure and local attention mechanism. The improved model significantly enhances local texture modeling accuracy and overall classification robustness without increasing computational cost.

(1) Multi-channel input modeling based on Fusion–Sobel–Moments–Gabor

Wood cross-sections exhibit significant anisotropy and multi-scale texture features. A single modality is insufficient to simultaneously describe complex structures such as vessel outlines, ray distribution, and periodic textures. Therefore, TextureFormer constructs four-modal complementary features at the input and stacks them along the channel dimension to form a 12-channel tensor $X^{mm}\in R^{H\times W\times 12}$:

(14) $$X^{mm}=concat(X^{\left(f\right)},X^{\left(s\right)},X^{\left(m\right)},X^{\left(g\right)})$$

This multimodal construction allows the model to explicitly obtain complementary information on “global layering, edge orientation, geometry, and periodic texture” at the input layer, providing rich priors for subsequent fusion and attention allocation. After normalization and alignment, all modalities correspond completely in space, providing geometric consistency for subsequent interest point guidance.

(2) Texture Stem: Lightweight Blending and Channel Recalibration

**Figure 6:**
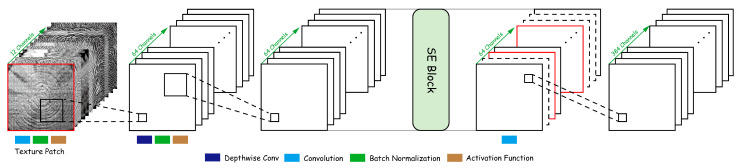
Texture Stem module. The red solid squares and dashed squares illustrate the channel-wise feature excitation and recalibration introduced by the SE module, reflecting the enhancement and adjustment of channel feature responses.

To achieve high-quality fusion of 12-channel features without reducing spatial resolution, this paper designs the Texture Stem module shown in Figure 6.

(15) $$F_{stem}={Conv}_{1\times 1}(SE(\sigma_{a}(BN(DWConv(\sigma_{a}(BN(Conv(X^{mm}))))))))$$

- Local fusion: The first layer of 3×3 convolution mixes the 12 modal channels into a 64-dimensional feature space, completing the initial coupling of multi-source features:
  (16) $$X_{1}=\sigma_{a}(BN({Conv}_{3\times 3}(X^{mm})))\in R^{H\times W\times 64}$$

- Spatial refinement: Depthwise separable $DWConv_{3\times 3}$ convolution enhances spatial consistency while maintaining low parameter count:
  (17) $$X_{2}=\sigma_{a}(BN({DWConv}_{3\times 3}(X_{1})))$$

- Channel recalibration via SE mechanism:
  (18) $$w=\sigma \left(W_{2}\delta \left(W_{1}GAP(X_{2})\right)\right),{\;X}_{3}=X_{2}⊙w$$

- High-dimensional embedding: The final 1×1 convolution increases the feature dimension to embed_dim=384:
  (19) $$T\in R^{H\times W\times 384}$$

(3) Interest-guided group attention mechanism

Although the Texture Stem fuses 12-channel multimodal texture features into a unified high-dimensional representation, the translation invariance of convolutional operations tends to weaken or even eliminate the spatial saliency distributions of different texture modalities. As a result, the model may fail to preserve the *key spatial locations* of structural texture features, such as edge orientations, frequency responses, and geometric abrupt changes. Therefore, multimodal texture fusion performed solely in the pixel domain is insufficient to explicitly model spatial saliency information that is critical for structural discrimination.

To compensate for the lack of explicit spatial saliency priors in convolution-based feature fusion, we propose a Modality-Guided Grouped Multi-Head Attention (MG-GMH) mechanism to explicitly model the spatial importance of key texture regions. Rather than encoding texture content itself, MG-GMH introduces structured spatial saliency constraints to guide the attention mechanism toward regions that are more critical for wood structural discrimination.

(1) Interest-Based Saliency Modeling

For each sample, candidate interest points are extracted independently from four texture modalities, namely, fusion texture, Sobel, moments, and Gabor features. Each interest point is represented as $\left(x_{i},y_{i},s_{i}\right)$, where $\left(x_{i},y_{i}\right)$ denotes the spatial coordinate and $s_{i}\in [0,1]$ denotes the original confidence score. To jointly consider confidence strength and spatial dispersion, a True Score is defined as

(20) $$T_{i}=\alpha s_{i}+(1-\alpha )\frac{1}{K_{i}}\sum_{j\in S_{i}}\;min\left(1,\frac{d_{ij}}{L}\right)$$

where $d_{ij}$ is the Euclidean distance between point i and a previously selected point j, L is a normalization constant, and α∈[0,1] controls the trade-off between confidence and spatial diversity. Based on the True Score, a fixed number of interest points are selected for each modality.

The selected interest points are then projected onto the Transformer patch grid with stride p, and a sparse token-level saliency map is constructed as

(21) $$t_{x}=\left\lfloor \frac{x_{k}}{p}\right\rfloor ,\;{\;t}_{y}=\left\lfloor \frac{y_{k}}{p}\right\rfloor ,\;\;\;H^{\left(m\right)}[t_{y},t_{x}]=max\left(H^{\left(m\right)}[t_{y},t_{x}],T_{k}\right)$$

where $H^{\left(m\right)}$ denotes the token-level saliency distribution of modality m. This process aligns interest points from continuous spatial coordinates to discrete Transformer tokens, providing modality-specific saliency priors for subsequent attention guidance.

(2) Modality-Based Attention Head Grouping

To prevent saliency interference across different texture modalities, MG-GMH introduces a modality-level grouping constraint at the attention head level. Given an attention head index h, its corresponding modality is determined as

(22) h∼mod∼4∈{Fusion, Sobel, Moments, Gabor}

This grouping strategy ensures that each group of attention heads is guided exclusively by the saliency prior of its assigned modality, thereby achieving modality-specific structural decoupling within the attention mechanism.

(3) Saliency Bias Injection

Within each attention window, the token-level saliency vector of modality m is denoted as

(23) $$h^{\left(m\right)}=(h_{1}^{\left(m\right)},...,h_{N}^{\left(m\right)})$$

An additive saliency bias matrix is constructed as

(24) $$B^{\left(h\right)}[i,j]=h_{j}^{\left(m\right)}$$

and injected into the attention logits of the h-th attention head:

(25) $$A_{logit}^{\left(h\right)}=\frac{QK^{⊤}}{\sqrt{d}}+RPB+B^{\left(h\right)}$$

**Figure 7:**
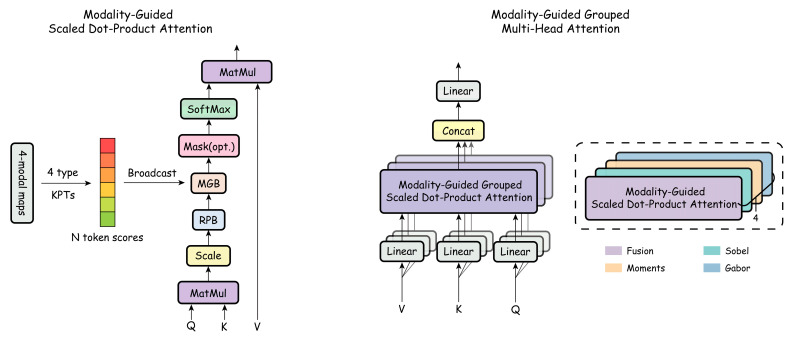
Multimodal keypoint-guided attention mechanism. The color bar indicates the relative magnitude of the saliency bias $B_{i}$, where warmer colors represent higher saliency values.

The bias is broadcast along the key dimension, allowing tokens with higher saliency values to receive larger attention weights after normalization. In this manner, MG-GMH explicitly guides the attention mechanism to focus on structurally salient texture regions, as illustrated in Figure 7.

### 3.4. Complementary and Collaborative Learning Training

#### 3.4.1. Method Overview

To fully leverage the complementarity of SpectralFormer++ and TextureFormer, this section adopts the complementary collaborative learning paradigm ST-former, which trains only the inter-class fusion weights. After the two branches have fully converged on their respective data and tasks, their parameters are fixed, and only the one-dimensional fusion coefficient set $\{\lambda_{c}{\}}_{k=1}^{K}$ for each class is trained to complete the subsequent complementary fusion.

Given the spectral branch logit $T_{k}^{\left(s\right)}$ and the texture branch logit $T_{k}^{\left(t\right)}$ of class k, the fused logit is defined as

(26) $${\tilde{T}}_{k}=(1-\sigma_{s}(\lambda_{c}))T_{k}^{\left(s\right)}+\sigma_{s}(\lambda_{c})T_{k}^{\left(t\right)}$$

#### 3.4.2. Training Objective and Loss Function

Only $\left\{\lambda_{c}\right\}$ are updated by minimizing the cross-entropy loss:

(27) $$L_{CE}=-\frac{1}{B}\sum_{i=1}^{B}\;log\frac{exp({\tilde{T}}_{y_{i}})}{\sum_{k=1}^{K}\;exp({\tilde{T}}_{k})}$$

To prevent $\sigma_{s}(\lambda_{c})$ from collapsing to extreme values (i.e., overly biased toward 0 or 1), a mild regularization term is introduced:

(28) $$L_{reg}=\frac{1}{K}\sum_{c=1}^{K}\;(\sigma_{s}(\lambda_{c})-0.5{)}^{2}$$

The final objective is as follows:

(29) $$L=L_{CE}+\beta L_{reg},\beta \in [10^{-4},10^{-2}]$$

In practice, we found that $\beta =5\times 10^{-3}$ stabilizes training while avoiding suppression of personalized bias. If the majority of $\sigma_{s}(\lambda_{c})\;$ values approach approximately 0.5 after training, β can be appropriately decreased.

## 4. Experiment and Results

### 4.1. Experimental Analysis of Wood Spectral Space Construction

#### 4.1.1. Experimental Analysis of Hyperspectral Band Selection

**Figure 8:**
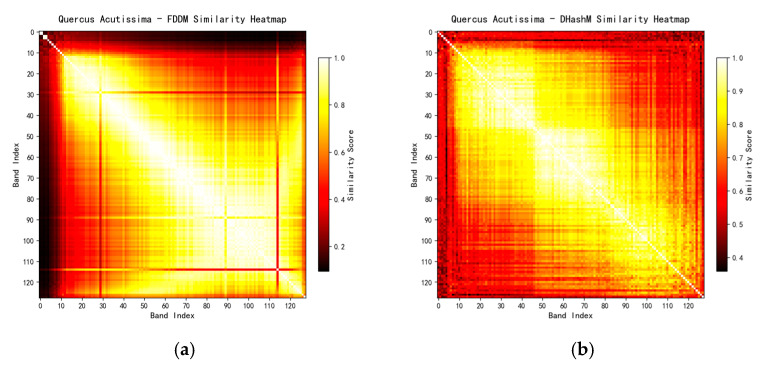

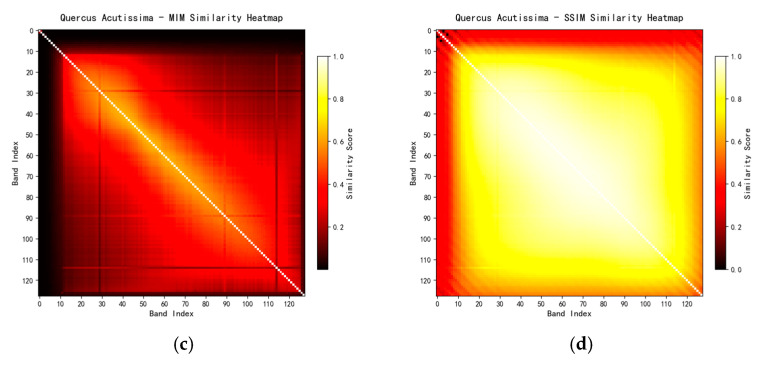
Similarity heatmap of *Q. aliena* Blume based on four types of indicator bands: (**a**) FDDM; (**b**) DHashM; (**c**) MIM; (**d**) SSIM.

Taking the hyperspectral cube of the cross-section of *Quercus aliena* Blume wood as an example, similarity matrices are first calculated via four types of metrics (FDDM, DHashM, MIM, SSIM), and heatmaps are generated, as shown in Figure 8.

**Figure 9:**
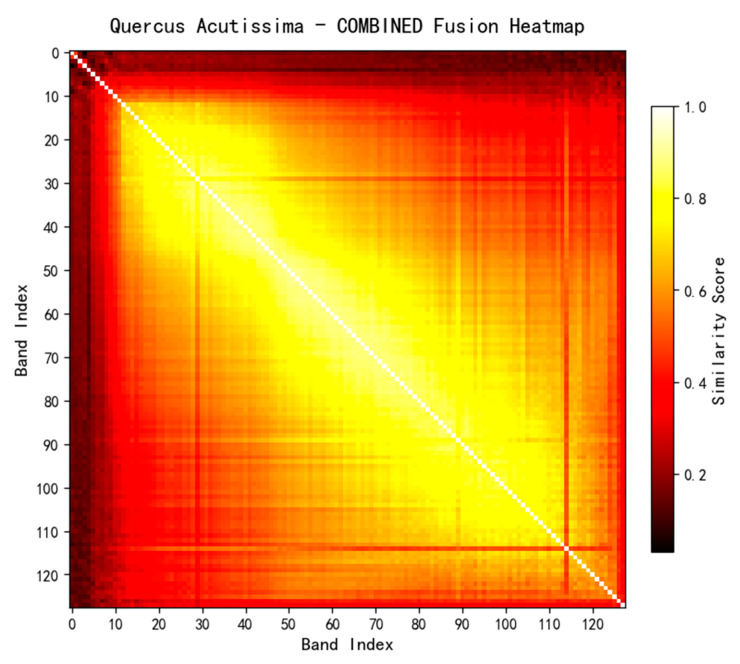
Fusion heatmap of *Q. aliena* Blume based on the integrated similarity matrix.

In this study, the equal weighting method is employed to allocate weights to various evaluation indicators, specifically assigning identical weight coefficients to the four core indicator categories. This weighting design aims to ensure that the characteristic attributes of each dimension receive equally important consideration during the analysis, $w_{m}=0.25$ thereby preventing features of certain dimensions from being weakened or exaggerated in the final evaluation results due to weight discrepancies. With this balanced weight allocation strategy, the contribution of features from different dimensions to the overall evaluation system can be maintained relatively balanced, making the final evaluation results more comprehensive and objective, as shown in Figure 9.

The multi-strategy band selection mechanism applies appropriate weighting to uncovered spectral regions based on differences, ensuring that the selected bands are more evenly distributed across the full spectrum and provide more comprehensive information. Finally, the top 10 band pairs with the lowest similarity are extracted from the comprehensive similarity matrix, as shown in Table 2.

Overall, the bands selected by Strategy A are relatively evenly distributed across the visible, red, and near-infrared regions, effectively reflecting the main reflection characteristics of wood in different bands. In contrast, Strategy B, due to the introduction of a coverage reward factor, yields selected bands that are more dispersed across the full spectrum, with stronger information complementarity.

#### 4.1.2. Experimental Analysis of Wavelet Transform Band Fusion

**Figure 10:**
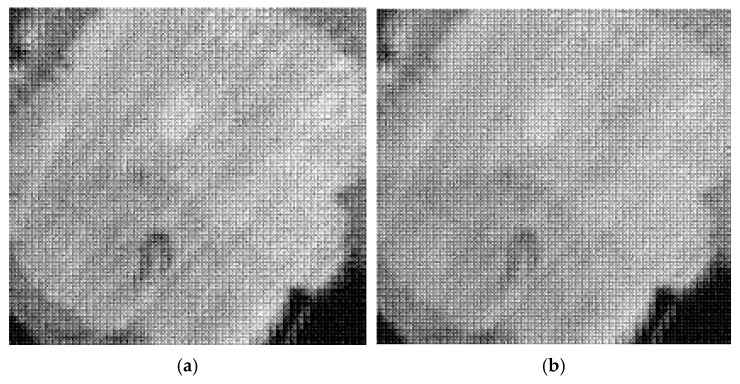
Comparison of wavelet fusion images under different strategies: (**a**) Strategy A; (**b**) Strategy B.

During image reconstruction, the fused subband set $\left(A^{F},\{H^{F},V^{F},D^{F}\}\right)$ is processed via inverse wavelet transform to obtain a single fused image $I^{F}$. To eliminate brightness differences between various samples and improve visualization effects, the fusion results were subjected to linear normalization. The results are converted to uint8 format, with the pixel range normalized to [0,255]. Finally, fused images are generated according to Strategies A and B, respectively, as shown in Figure 10.

#### 4.1.3. Experimental Analysis of Spectral Feature Extraction

**Figure 11:**
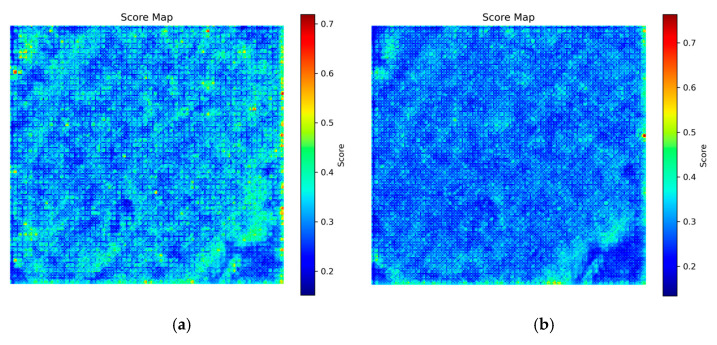
Multi-feature fusion evaluation score maps of Strategies A and B: (**a**) Strategy A; (**b**) Strategy B.

To avoid the influence of a single fusion scheme on subsequent detection, the wavelet fusion results of Strategies A and B are, respectively, enhanced and subjected to multi-feature weighted evaluation, yielding two score maps, $S^{\left(A\right)}(x,y)$ and $S^{\left(B\right)}(x,y)$, as shown in Figure 11.

**Figure 12:**
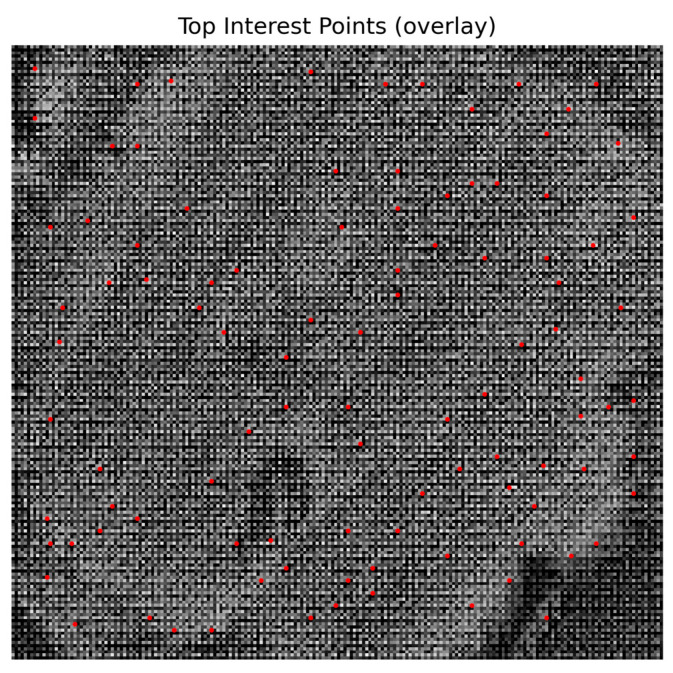
Detected interest points overlaid on the multi-feature evaluation map.

It should be noted that the Harris corner response is not only used as a corner strength feature for weighted calculation in the multi-feature score map but also employed for generating candidate points during the interest point extraction stage. The former reflects the saliency of local intensity changes, while the latter determines candidate locations; the two cooperate with each other to achieve high-precision interest point detection, as shown in Figure 12.

**Figure 13:**
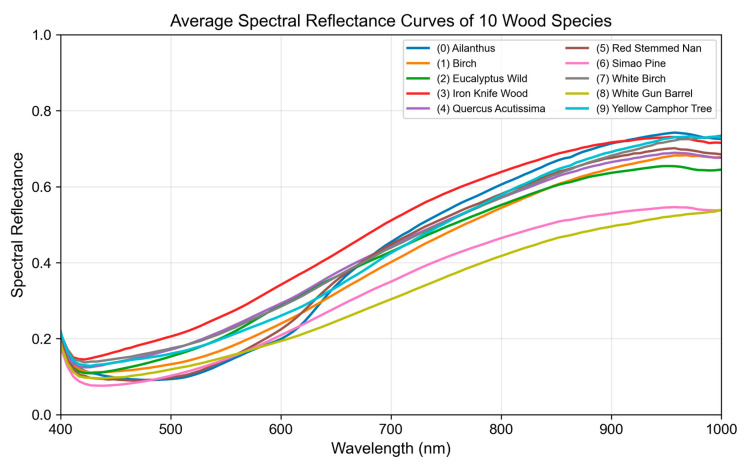
Average spectral reflectance curves of ten representative wood species across all heights.

Finally, the 128-dimensional reflectance spectral features of the corresponding pixels are extracted from the original hyperspectral image at the interest point locations. The resulting spectral features possess strong physical interpretability and can reflect differences in the spectral responses of different wood components. Figure 13 shows the average spectral reflectance curves of ten tree species.

### 4.2. Experimental Analysis of Wood Texture Space Construction

**Figure 14:**
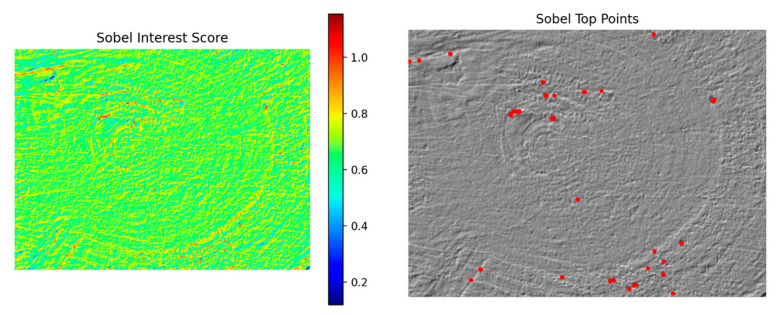
Score distribution map and detected interest points of the Sobel modality.

The Sobel modality can effectively characterize structural features such as wood pores, fiber boundaries, and vessel distributions. As shown in Figure 14, the left panel displays the multi-feature score distribution map of the Sobel modality, while the right panel presents the corresponding interest point detection results.

**Figure 15:**
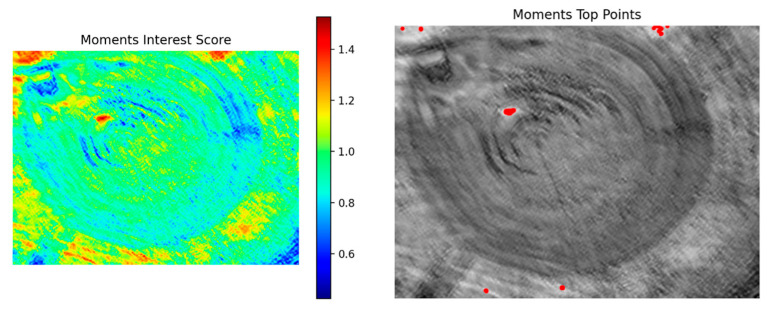
Score distribution map and detected interest points of the Moments modality.

The Moments modality characterizes the local texture energy distribution and shape orientation through second-order geometric moments, effectively capturing the scale differences and geometric configuration features of wood structure. As shown in Figure 15, the left image shows the score distribution map of the Moments modality, and the right image shows the results of its interest point detection.

**Figure 16:**
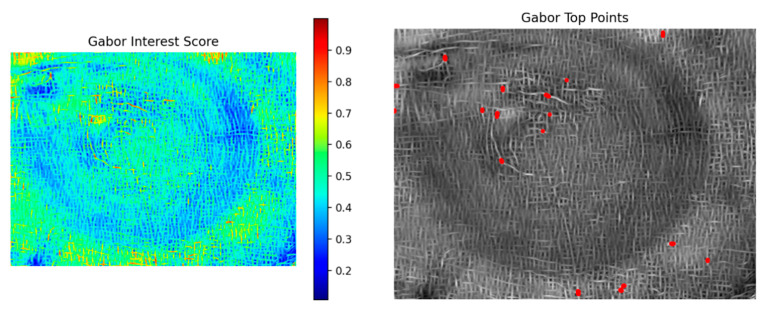
Score distribution map and detected interest points of the Gabor modality.

The Gabor modality reflects the directional and frequency characteristics of textures and can effectively characterize the periodic radial distribution of wood textures. As shown in Figure 16, the left panel shows the multi-feature score distribution map of the Gabor modality, while the right panel shows the corresponding interest point detection results; the interest points are mainly concentrated in the high-frequency regions of texture energy.

### 4.3. Experimental Analysis of Single Model Training

(1) Training and Performance Evaluation of the SpectralFormer++

**Figure 17:**
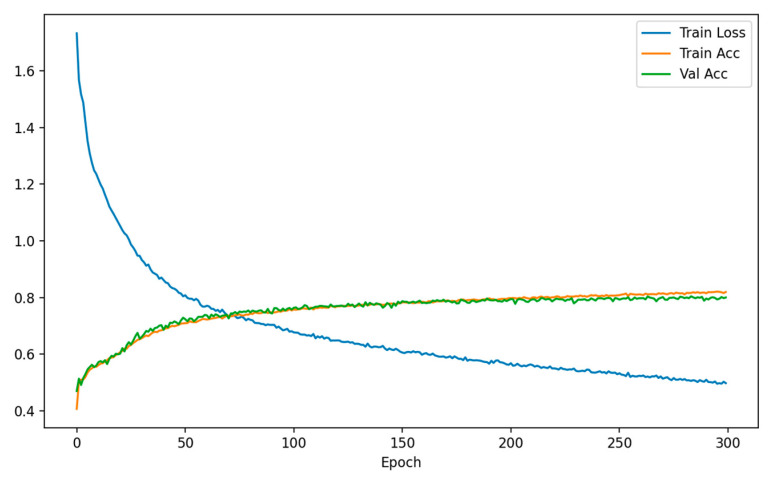
Training loss and accuracy curves of the SpectralFormer++ model.

The AdamW optimizer (learning rate $1\times 10^{-4}$), batch size = 256, and cross-entropy loss function are adopted for end-to-end training, with the number of training epochs set to 300. As shown in Figure 17, the training loss continuously decreases, while the training accuracy and validation accuracy steadily increase and tend to converge in the later stages, indicating that the model exhibits a stable training process and excellent generalization performance.

**Figure 18:**
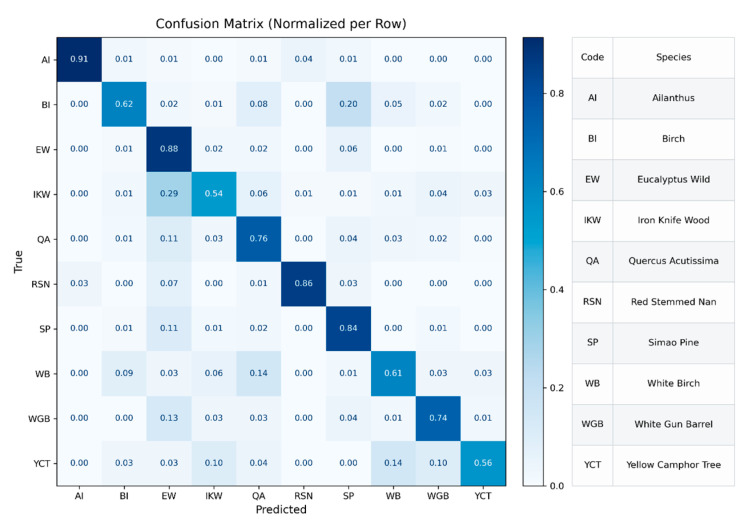
Row-normalized confusion matrix of the SpectralFormer++ model on the test set.

On the test set, SpectralFormer++ achieves an overall classification accuracy of 81.55%. As shown in Figure 18 (confusion matrix), categories such as Ailanthus, Eucalyptus Wild, and Red-Stemmed Nan exhibit high recognition accuracy on the diagonal, indicating that the model has excellent discriminative ability for tree species with distinct spectral features. However, for woods with similar spectral characteristics, such as Iron Knife Wood and Birch, White Birch, White Gun Barrel and Yellow Camphor Tree, a certain degree of confusion still exists, with their diagonal accuracies ranging from 0.54 to 0.74. This is mainly due to the highly similar reflectance characteristics of these tree species in the visible and near-infrared bands.

(2) Training and Performance Evaluation of the TextureFormer

**Figure 19:**
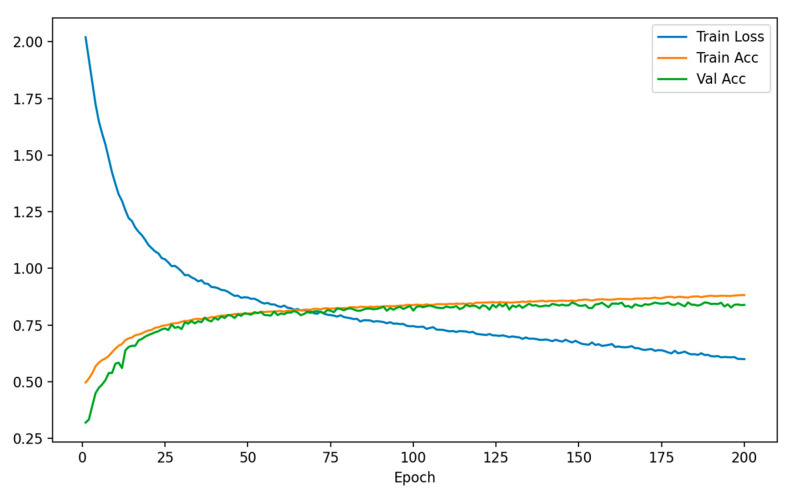
Training loss and accuracy curves of the TextureFormer model.

To verify the effectiveness of the TextureFormer in multimodal texture feature recognition tasks, training and testing are conducted on the fused multimodal texture dataset. The AdamW optimizer (learning rate $3\times 10^{-4}$, batch size = 32, number of training epochs = 300) is adopted. During training, the model input is a 12-channel texture stacked image composed of four modalities (Fusion, Sobel, Moments, and Gabor), combined with interest point-guided coordinates as auxiliary input. As shown in Figure 19, the model’s loss continuously decreases during training, while the accuracy improves rapidly in the early stages of iteration and enters a stable phase after approximately 100 epochs.

**Figure 20:**
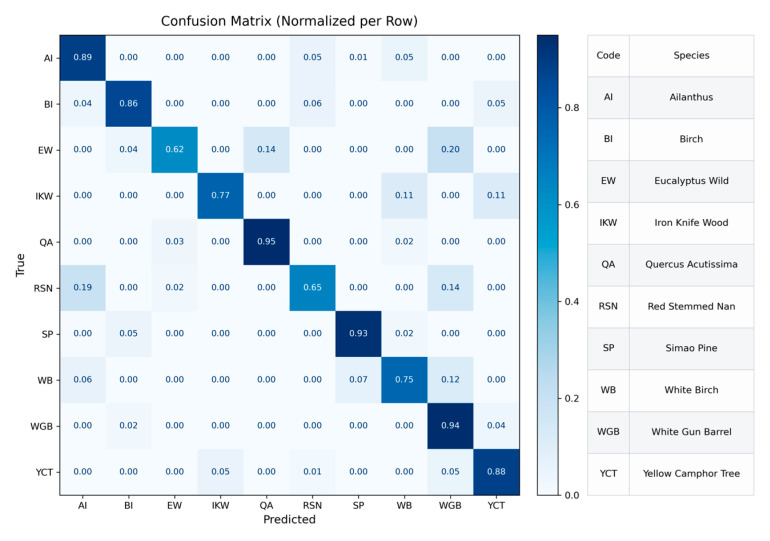
Row-normalized confusion matrix of the TextureFormer model on the test set.

On the test set, the overall classification accuracy of the TextureFormer model reaches 86.27%. Its confusion matrix is shown in Figure 20, demonstrating that the model performs well overall in recognition. From the figure, it can be seen that Quercus acutissima, White Gun Barrel, and Simao Pine achieved high classification accuracy, indicating that the model could effectively learn the key features of these tree species with distinctly different texture structures. However, there was still some confusion among certain samples. Particularly, categories such as Red Stemmed Nan, Eucalyptus wild, and White Birch—characterized by similar textures or grayscale distributions—showed relatively lower accuracy, indicating that the model still faced difficulty in distinguishing these subtle texture differences.

### 4.4. Complementary Collaborative Learning Parameter Training and Performance Evaluation

**Figure 21:**
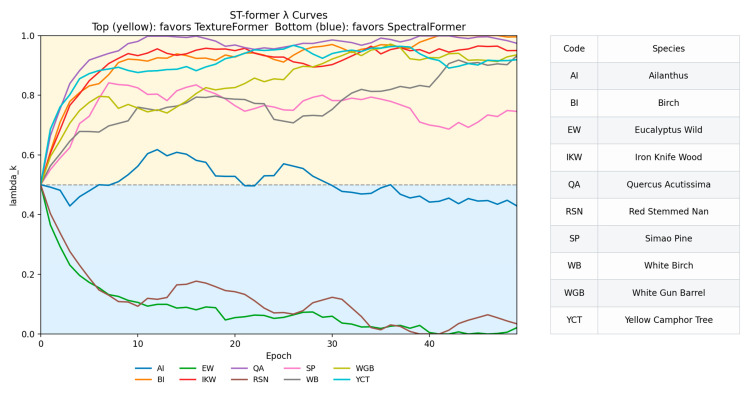
Convergence curves of category-wise fusion parameters $\lambda_{k}$.

Figure 21 shows the $\lambda_{k}$ evolution curves of ten major tree species. The results indicated that all categories of $\sigma_{s}(\lambda_{k})$ experienced a phase of rapid fluctuation at the beginning of training, followed by gradual convergence and stabilization in different ranges, with the overall distribution concentrated between 0.4 and 0.6. This suggested that the model achieves a relatively balanced weight allocation between the spectral and texture modalities. Among them, the $\lambda_{k}$ values for Eucalyptus wild, Red Stemmed Nan, and other species are slightly lower, indicating that the model tends to rely more on SpectralFormer++ when distinguishing woods with prominent spectral features, whereas the higher $\lambda_{k}$ values for Yellow Camphor Tree, Betula, and similar samples suggest that their recognition mainly depends on the texture branch TextureFormer. Overall, the convergence of $\lambda_{k}$ confirms that the strategy of training only the fusion parameters effectively avoids overfitting and oscillation issues and provides good interpretability at the category level.

**Figure 22:**
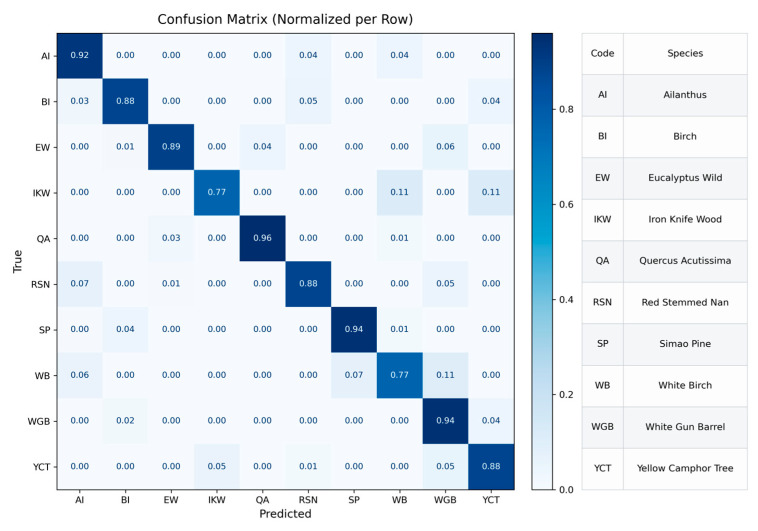
Row-normalized confusion matrix of the TextureFormer model on the test set.

The test results of the joint model are shown in Figure 22. Compared with single-modality models, the diagonals of the row-normalized confusion matrix is more concentrated, and the overall classification accuracy increases to 92.27%. For categories such as Ailanthus and Quercus acutissima, the diagonal values exceed 0.9, indicating that the model can fully utilize the complementary information of spectral morphology and texture structure to achieve high-confidence discrimination. For categories like Iron Knife Wood and White Birch—characterized by similar textures and adjacent spectral features—partial confusion still exists, but their error rates are significantly reduced compared with single-modality models.

In addition, classes such as Birch, White Gun Barrel, and Simao Pine, which are easily confused in single-branch models, show significant improvement after joint training, with the proportion of off-diagonal elements reduced by more than half, indicating that the adaptive weight adjustment mechanism effectively enhances the efficiency of modality collaboration. Overall, the complementary collaborative model significantly improves the model’s generalization capability and classification robustness while considering both spectral and texture features, providing reliable experimental support for subsequent multimodal wood recognition. In summary, the ST-former model, through learnable inter-class fusion weights, effectively balances the importance of spectral and texture features, not only improving overall recognition accuracy but also maintaining interpretability and stability in the model structure, offering a reliable fusion approach for future multimodal wood recognition.

## 5. Discussion

The research method of this article is the perfect cross application of computer vision, spectroscopy, and artificial intelligence in wood science. This research method has unique advantages over wood species identification methods such as NIRS, stable isotopes and mineral elements, DNA, etc. Compared with the NIRS method [19,45,46], the method studied in this paper can learn the most discriminative microscopic features from the hyperspectral images of wood cross-sections, with higher recognition accuracy and more robust models. Compared with stable isotope and mineral element methods [24,47,48], the research method in this paper can establish a complex nonlinear mapping model between isotope/element data and tree species and origin, which can explore deeper and more complex patterns and achieve more accurate identification of wood species. Compared with the DNA method [32,49,50], the research method in this article is completely non-destructive and has a faster testing time. However, DNA sampling usually requires drilling or cutting a small amount of wood chips for testing.

The classification results shown in Figure 22 indicate that, even under the spectral–texture joint modeling framework, noticeable confusion still exists among certain tree species, suggesting that the joint model retains inherent limitations when distinguishing specific categories. Further analysis reveals that species with lower classification accuracy generally exhibit highly similar spectral reflectance curve shapes, while their cross-sectional textures show only subtle differences in vessel distribution, directional patterns, and structural scale. Under such conditions, neither spectral nor texture modalities can provide sufficiently discriminative information, and multimodal fusion alone is unable to fundamentally overcome the limited separability of the original features. As a result, the performance improvement of the joint model for highly similar categories remains constrained.

From a modeling perspective, the current joint framework primarily relies on one-dimensional spectral sequences and two-dimensional cross-sectional texture features for classification. While this design is effective for many tree species, it imposes an upper bound on representational capacity when involving species that are highly similar in structure and composition. In particular, higher-level structural differences within wood, such as cell-scale organization, micro-anatomical characteristics, or three-dimensional spatial distributions, are not explicitly modeled in the current approach. This limitation further restricts the discriminative capability of the joint model for certain species.

In addition, environmental factors associated with tree growth are not incorporated into the current recognition framework, although such factors may introduce discriminative information that is not captured by spectral and texture features alone. Previous studies have shown that growth environment conditions, including climate factors, temperature variation, precipitation, and soil nutrient availability, can influence wood formation processes and are partially reflected in wood structural and material characteristics [21,51]. For tree species with highly similar spectral and texture features, variations induced by growth environments may provide supplementary cues that help reduce inter-class ambiguity.

Based on the above analysis, future work aimed at improving classification accuracy across all categories—particularly for highly similar species—may consider extending the current spectral–texture joint framework in several directions. First, incorporating samples collected from diverse growth regions and environmental conditions could enhance the model’s ability to capture intra-class variability. Second, integrating higher-resolution texture information, micro-anatomical features, or three-dimensional structural representations may help compensate for the limitations of one-dimensional spectral and two-dimensional texture features. Finally, environmental parameters such as climatic and nutrient indicators could be introduced as sample-level conditional information or as auxiliary constraints at the decision stage, thereby complementing existing features from a growth-mechanism perspective and improving discrimination among highly similar tree species.

## 6. Conclusions

This paper addresses the issues associated with traditional wood identification methods, such as low efficiency, susceptibility to noise interference, and difficulty in distinguishing similar tree species, and proposes an intelligent wood species identification method based on the integration of multimodal texture-dominant features and deep learning. Based on hyperspectral imaging data, this method constructs deep feature representations from two perspectives (spectral domain and texture domain) and combines learnable modal fusion weights to achieve the unity of high precision and interpretability in wood recognition. In terms of spectral feature modeling, this paper designs the SpectralFormer++ model and introduces a spectral derivative prior and a front-end embedding normalization mechanism, effectively alleviating the instability caused by band redundancy and amplitude differences and improving the morphological expression ability of spectral features and the convergence stability of the model. For texture feature modeling, the TextureFormer model is proposed, which fully captures the fine-grained texture structures and directional patterns of wood cross-sections through a fusion-based Texture Stem and a grouping attention mechanism for multimodal input. Based on the output results of the two models, the ST-former complementary collaborative fusion framework is further constructed, and inter-class adaptive weight learning is realized, enabling the model to dynamically balance the contributions of spectral and texture features for different wood categories.

The experimental results show that the overall classification accuracy of the proposed joint model on 10 typical wood datasets from Yunnan reaches 90.27%, which is approximately 8% higher than that of single-modal models. Among these species, *Eucalyptus rudis* Endl, *Phoebe rufescens* H. W. Li, and other tree species with significant texture differences achieve the highest recognition accuracy, while *Betula platyphylla* Sukaczev, *Senna siamea* (Lam.) H. S. Irwin & Barneby, and other tree species with similar spectra exhibit a significantly lower confusion rate. This proves the effectiveness of the complementary synergistic mechanism in feature fusion and modal decoupling. The inter-class distribution of parameters further elucidates the model’s interpretability: the values for texture-dominated tree species are significantly higher than those for spectrum-dominated classes, indicating that the model can adaptively learn the feature dependencies of different tree species.

In general, the spectral–texture collaborative learning framework proposed in this paper achieves a balance between recognition accuracy, stability, and interpretability for wood species recognition tasks and provides a novel and promising idea for multimodal intelligent recognition with potential for application in the field of hyperspectral wood recognition. Future work can extend this framework to larger-scale datasets with more tree species and integrate 3D spectral structures or microstructural images to develop a more versatile and robust wood recognition system.

## Figures and Tables

**Table 1:** Comparison of identification methods for wood species.

Method	Characteristic Markers	Advantage	Disadvantage
NIRS	Detection of absorption spectra of molecules in the near-infrared region.	This method has the characteristics of fast, non-destructive, and efficient.	Difficult to identify characteristic chemical substances, interference caused by overlapping adjacent chromatographic peaks affects the detection results.
Stable Isotope	Isotopic ratio	High success rate of origin identification and minimal pollution.	δ13C, δ2H, δ^18^O, etc., are susceptible to interannual/seasonal driven radial fractionation interference; high requirements for instruments and equipment; the database of stable isotopes in wood is lacking.
Mineral Elements	Feature element	There are many types of candidate elements and high throughput.	The process of selecting feature element tags is cumbersome and labor-intensive; easy to be disturbed by artificial agricultural activities such as fertilizer application; the database of mineral elements in wood is lacking.
DNA	Characteristic gene fragments	Easy to classify, good repeatability, high stability, and less susceptible to environmental interference.	High quality DNA from deep processed wood products is difficult to obtain stably.

**Table 2:** Lowest-similarity top 10 band pairs of *Q. aliena* Blume.

Strategy A	Strategy B
Band_8 (409.24 nm)	Band_8 (409.24 nm)
Band_11 (424.97 nm)	Band_12 (430.22 nm)
Band_16 (451.20 nm)	Band_16 (451.20 nm)
Band_58 (671.46 nm)	Band_40 (577.06 nm)
Band_64 (702.93 nm)	Band_67 (718.66 nm)
Band_69 (729.15 nm)	Band_111 (949.42 nm)
Band_72 (744.88 nm)	Band_115 (970.39 nm)
Band_115 (970.39 nm)	Band_120 (996.61 nm)
Band_125 (1022.84 nm)	Band_124 (1017.59 nm)
Band_128 (1038.57 nm)	Band_127 (1033.33 nm)

## Data Availability

The data underlying this article will be shared on reasonable request to the corresponding authors. The data are not publicly available due to various data sources that have been introduced in the paper.

## Abbreviations

The following abbreviations are used in this manuscript:

AI	Ailanthus	*Ailanthus altissima* (Mill.) Swingle
BI	Birch	*Betula alnoides* Buch.-Ham. ex D. Don
EW	Eucalyptus Wild	*Eucalyptus rudis* Endl
IKW	Iron Knife Wood	*Senna siamea* (Lam.) H. S. Irwin & Barneby
QA	Quercus Acutissima	*Quercus aliena* Blume
RSN	Red Stemmed Nan	*Phoebe rufescens* H. W. Li
SP	Simao Pine	*Pinus kesiya* var. *langbianensis* (A. Chev.) Gaussen
WB	White Birch	*Betula platyphylla* Sukaczev
WGB	White Gun Barrel	*Fraxinus malacophylla* Hemsl
YCT	Yellow Camphor Tree	*Cinnamomum parthenoxylon* (Jack) Meisn.
Symbol	Description	
i,j	Indices of spectral bands	
$S_{ij}^{comb}$	Comprehensive similarity between spectral bands i and j, obtained by weighted fusion of multiple indicators	
$\Delta_{ij}$	Spectral difference between band iand band j$,\;\mathrm{defined}\;\mathrm{as}\;\Delta_{ij}=1-S_{ij}^{comb}$	
Ω	Set of selected spectral bands	
b	Index of a candidate spectral band	
$b^{*}$	Selected band maximizing the minimum distance to the current band set	
k	Target number of selected spectral bands	
$\lambda_{cov}$	Coverage reward factor in the coverage-aware Max–Min band selection strategy	
$r\left(b\right)$	Spectral region (VIS, RED, or NIR) to which band b belongs	
C	Set of spectral regions already covered by the selected band set	
$I_{band}$	Hyperspectral image cube after band filtering	
B	Number of spectral bands after selection	
H,W	Spatial height and width of the hyperspectral image	
$I_{band,b}$	Image corresponding to the b-th selected spectral band	
$N_{band}$	$\mathrm{Number}\;\mathrm{of}\;\mathrm{selected}\;\mathrm{band}\;\mathrm{images}\;\mathrm{used}\;\mathrm{for}\;\mathrm{fusion}\;(N_{band}=10$)	
L	Decomposition level of the wavelet transform	
db4	Daubechies-4 wavelet basis function	
$I_{fuse}(x,y)$	Input fused image in the spatial domain	
F(u,v)	Fourier transform of the input image	
$\left(u_{i},v_{i}\right)$	Center coordinates of the i-th periodic interference peak in the frequency domain	
M(u,v)	Gaussian notch filter mask in the frequency domain	
$\alpha_{n}$	Suppression intensity of the notch filter	
$\sigma_{n}$	Radius of the Gaussian notch filter	
$I_{dn}(x,y)$	Image after notch filtering and inverse Fourier transform	
$\gamma_{h}$	Gamma correction parameter used in local enhancement	
$C\in R^{H\times W\times B}$	Original hyperspectral image cube	
$I_{pca}$	Grayscale base image obtained from the first principal component of the hyperspectral cube	
$G_{x},G_{y}$	Horizontal and vertical Sobel gradient responses	
$F_{sobel}$	Two-channel Sobel edge feature map	
N(⋅)	Linear normalization operator applied to feature response maps	
w	Window size for local geometric moment computation	
$k_{20},k_{02},k_{11}$	Second-order geometric moment kernels	
$M_{20},M_{02},M_{11}$	Second-order geometric moment response maps	
$F_{moments}$	Three-channel normalized geometric moment feature map	
$g\left(x,y;\theta \right)$	Gabor filter kernel at orientation θ	
θ	Gabor filter orientation angle	
$x^{\prime },y^{\prime }$	Rotated spatial coordinates in the Gabor filter	
λ	Wavelength of the Gabor filter	
$\sigma_{g}$	Standard deviation of the Gaussian envelope in the Gabor filter	
$\gamma_{g}$	Spatial aspect ratio of the Gabor filter	
$F_{gabor}$	Six-channel Gabor energy feature map	
$S_{kpt}(x,y)$	Multi-feature scoring map for texture saliency	
Harris	Harris corner response map	
Entropy	Local entropy feature map	
Sobel	Gradient magnitude feature map	
Laplace	Laplacian response map	
DoG	Difference-of-Gaussians response map	
SNR	Signal-to-noise ratio feature map	
t	Relative threshold for interest point detection	
d	Minimum spatial distance between interest points	
K	Number of selected interest points (K=100)	
$X^{sp}$	Three-channel spectral derivative input token consisting of original, first-order, and second-order spectral features	
W	Linear projection matrix in the spectral embedding layer	
b	Bias term of the spectral embedding layer	
d	Embedding dimension of the spectral token (d=64)	
E	Layer-normalized spectral embedding output	
LN(⋅)	Layer normalization applied to the spectral embedding	
$X^{\left(f\right)}$	Fusion-based texture feature map	
$X^{\left(s\right)}$	Sobel-based texture feature map	
$X^{\left(m\right)}$	Second-order geometric moment texture feature map	
$X^{\left(g\right)}$	Gabor energy-based texture feature map	
$X^{mm}$	Multimodal texture feature tensor concatenated along the channel dimension	
$F_{stem}$	Output feature map of the Texture Stem module	
$X_{1}$	Intermediate feature map after the first 3×3 convolution in the Texture Stem	
$X_{2}$	Intermediate feature map after depthwise separable convolution in the Texture Stem	
$X_{3}$	Channel-recalibrated feature map after SE attention	
w	Channel-wise attention weight generated by the SE mechanism	
T	High-dimensional texture embedding after the final 1×1$\mathrm{convolution}\;(T\in R^{H\times W\times 384}$)	
m	Modality index corresponding to fusion, Sobel, moments, or Gabor texture modality	
h	Attention head index in the multi-head attention mechanism	
$T_{i}$	True Score of the i-th interest point combining confidence strength and spatial dispersion	
$s_{i}$	Original confidence score of the i-th interest point	
$d_{ij}$	Euclidean distance between interest point iand a previously selected point j	
L	Normalization constant in the True Score computation	
α	Trade-off parameter controlling confidence strength and spatial dispersion	
p	Patch stride used to map spatial coordinates to Transformer tokens	
$H^{\left(m\right)}$	Token-level saliency map of modality m	
$h^{\left(m\right)}$	Token-level saliency vector of modality m within an attention window	
$B^{\left(h\right)}$	Modality-guided saliency bias matrix for the h-th attention head	
$A_{logit}^{\left(h\right)}$	Attention logits of the h-th attention head with saliency bias injection	
RPB	Relative position bias in window-based self-attention	
Q,K,V	Query, key, and value matrices in the attention mechanism	
$T_{k}^{\left(s\right)}$	Spectral branch logit of class k	
$T_{k}^{\left(t\right)}$	Texture branch logit of class k	
${\tilde{T}}_{k}$	Fused logit of class k	
$\lambda_{c}$	Class-wise fusion coefficient in complementary collaborative learning	
$\sigma_{s}(\cdot )$	Sigmoid function applied to fusion coefficients	
$L_{CE}$	Cross-entropy loss for fused logits	
$L_{reg}$	Regularization loss preventing fusion weight collapse	
L	Final training objective combining classification and regularization losses	
β	Weighting factor for the regularization term	
B	Batch size	
K	Number of classes	
$K_{i}$	$\mathrm{Number}\;\mathrm{of}\;\mathrm{selected}\;\mathrm{interest}\;\mathrm{points}\;\mathrm{in}\;S_{i}$$(i.e.,\;K_{i}=\left|S_{i}\right|$)	
i	Training sample index	
k	Class index	
c	Class index for fusion coefficient	
